# Supplementary material for: Involvement of PAR-2 in the Induction of Cell-Specific Matrix Metalloproteinase-2 by Activated Protein C in Cutaneous Wound Healing
Source: Int J Mol Sci. 2023 Dec 27;25(1):370. doi: 10.3390/ijms25010370 (PMC10779272; doi:10.3390/ijms25010370)
Supplement: Supplementary file 1 [file ijms-25-00370-s001.zip › ijms-2766299-supplementary.pdf]

Figure S1:

APC-stimulated wound healing was delayed in PAR-2 KO mice.

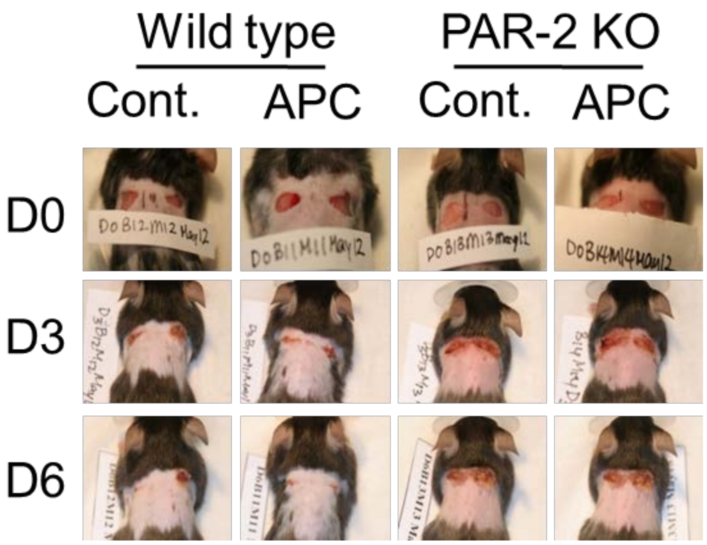

Representative photographs of skin wounds on days after PBS and APC treatment.

Figure S2:

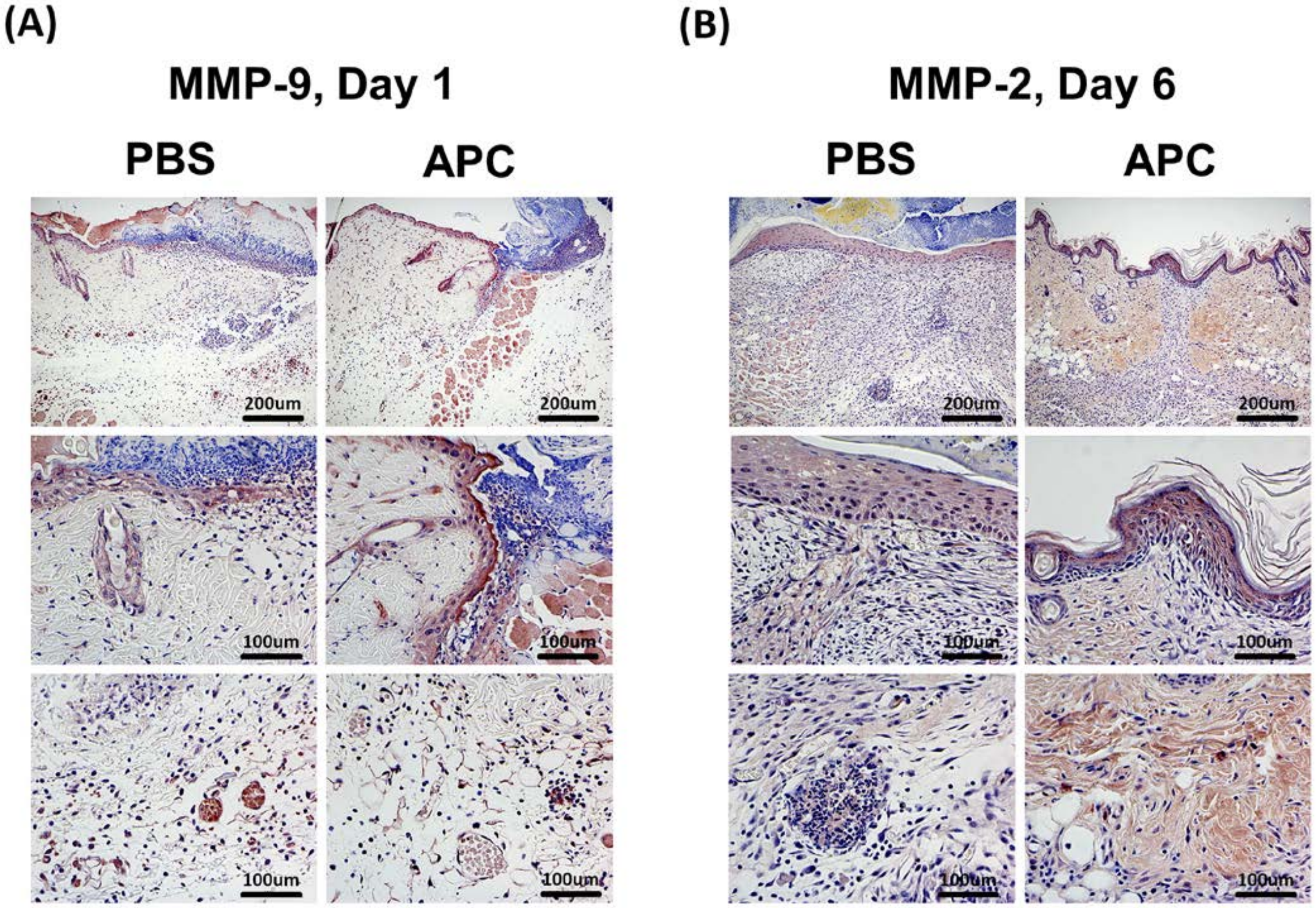

(A) Expression patterns of MMP-9 at day 1 (A) and MMP-2 at day 6 (B) by immunohistochemistry are shown to demonstrate the difference in staining level between PBS and APC-treated wounds. Scale bars, 200 μm (upper panels) and 100 μm (middle and lower panels).
